# Clinical and neuroimaging features of patients with claustrum sign

**DOI:** 10.3389/fneur.2025.1589940

**Published:** 2025-05-27

**Authors:** Chunyan Zhao, Meijiao Zhang, Qingping Zhang, Xinhua Bao

**Affiliations:** Department of Pediatrics, Peking University First Hospital, Beijing, China

**Keywords:** claustrum sign, neuroinflammation, febrile infection-related epilepsy syndrome, autoimmune encephalitis, myelin oligodendrocyte glycoprotein antibody-associated disease, Wilson’s disease, seizures, consciousness

## Abstract

**Objective:**

This study aimed at summarizing the clinical and neuroimaging features of patients with claustrum sign, so as to enhance the understanding of this imaging feature and explore its clinical significance.

**Methods:**

Clinical data from 20 patients with claustrum sign were collected. The clinical characteristics, neuroimaging evolution, and outcomes were analyzed.

**Results:**

This cohort included 14 males and 6 females and the median age of onset was 6 years old. Diagnoses included febrile infection-related epilepsy syndrome (FIRES) in 12 cases (60%), antibody-negative autoimmune encephalitis (AbNAE) in 4 (20%), myelin oligodendrocyte glycoprotein antibody-associated disease (MOGAD) in 3 (15%), and Wilson’s disease (WD) in 1 (5%). Predominant neurological symptoms included seizures (85%) and impaired consciousness (70%). The claustrum sign was observed on days 1–25 (median: day 11.5) after the onset of neurologic symptoms. It presented on the first MRI between days 1 and 14 (median: day 5.5) in 8 cases (40%), while it was absent on the first MRI (days 1–7, median: day 3) in the remaining 12 cases (60%) and appeared on the repeated MRIs (days 6–25, median: day 15). On the follow-up MRIs in 19 cases, the claustrum sign resolved on days 16–132 (median: day 53) in 17 patients, except one with AbNAE and one with WD. The patients with FIRES had the worst prognosis, all developed chronic epilepsy, 75% showed poor memory and calculation, and the median Pediatric Cerebral Performance Category (PCPC) score was 3. In contrast, patients with AbNAE and MOGAD had favorable outcomes with a median PCPC score of 1, respectively.

**Conclusion:**

The claustrum sign may represent a transient neuroinflammatory lesion and serve as an imaging marker of neuroinflammation. Lesions in the claustrum can lead to dysfunction of its connected regions, which could be one of the potential mechanisms underlying the high incidence of seizures and the impaired consciousness in children with this imaging feature. Long-term outcomes are closely related to the primary disease.

## 1 Introduction

The claustrum is a thin, irregular layer of gray matter located between the putamen and insular cortex, accounting for only 0.25% of the cerebral gray matter volume (1). The claustrum sign refers to hyperintense signals in the claustrum region on T2-weighted and fluid-attenuated inversion recovery (FLAIR) sequences of brain magnetic resonance imaging (MRI). This imaging feature was first reported in Wilson’s disease (WD) in 1993 (2), and subsequently reported in various neurological disorders including new-onset refractory status epilepticus (NORSE) (3–5), autoimmune encephalitis (AE), autoimmune-associated epilepsy (AAE) (6), febrile infection-related epilepsy syndrome (FIRES) (7–11), and acute necrotizing encephalopathy (ANE) (12). Currently, the pathophysiological mechanisms underlying the claustrum sign remain elusive. This study aims to summarize the clinical and neuroimaging features of patients with claustrum sign and explore its clinical significance.

## 2 Materials and methods

### 2.1 Participants

We retrospectively collected data from 20 children with claustrum sign on brain MRI who were admitted to the Department of Pediatrics, Peking University First Hospital between January 2020 and January 2024. The study was approved by the Ethics Committee of the Peking University First Hospital, China (No. 2024 scientific 506).

### 2.2 Clinical data collection and follow-up

The following clinical data from the patients with claustrum sign were collected during hospitalization/outpatient visits and followed up. (1) Demographic characteristics: age of onset, sex, and diagnosis; (2) Clinical data: prodromal symptoms, clinical manifestation including impaired consciousness status, seizures, treatment, and prognosis; (3) Ancillary tests: brain MRI (appearance and resolution time of the claustrum sign, concurrent lesions), cerebrospinal fluid (CSF), and electroencephalogram (EEG). All patients underwent brain MRI scans using a 3.0 T magnetic resonance scanner. The conventional MRI scanning sequences included T1-weighted imaging (T1WI), T2-weighted imaging (T2WI), fluid-attenuated inversion recovery sequence (FLAIR), and diffusion-weighted imaging (DWI). The last follow-up was conducted in December 2024. Neurological outcome was evaluated by the Pediatric Cerebral Performance Category (PCPC) scale (13), and graded as the follows: 1 (normal), 2 (mild disability), 3 (moderate disability), 4 (severe disability), 5 (coma/vegetative state), and 6 (brain death). PCPC score ≥ 2 after 6 months of onset was defined as neurological sequelae.

### 2.3 Statistical analysis

We used SPSS 27.0 (IBM Corp) for descriptive statistical summarization. Categorical data were shown as counts and percentages, and continuous data with a non-normal distribution were presented as median and range.

## 3 Results

### 3.1 Demographic characteristics

This study enrolled 20 patients with claustrum sign, including 14 (70.0%) males and 6 (30.0%) females. The median age of onset was 6 years old (range 3.2–12.6 years old). Previous developmental and family histories were unremarkable.

### 3.2 Neuroimaging features

**Figure 1:**
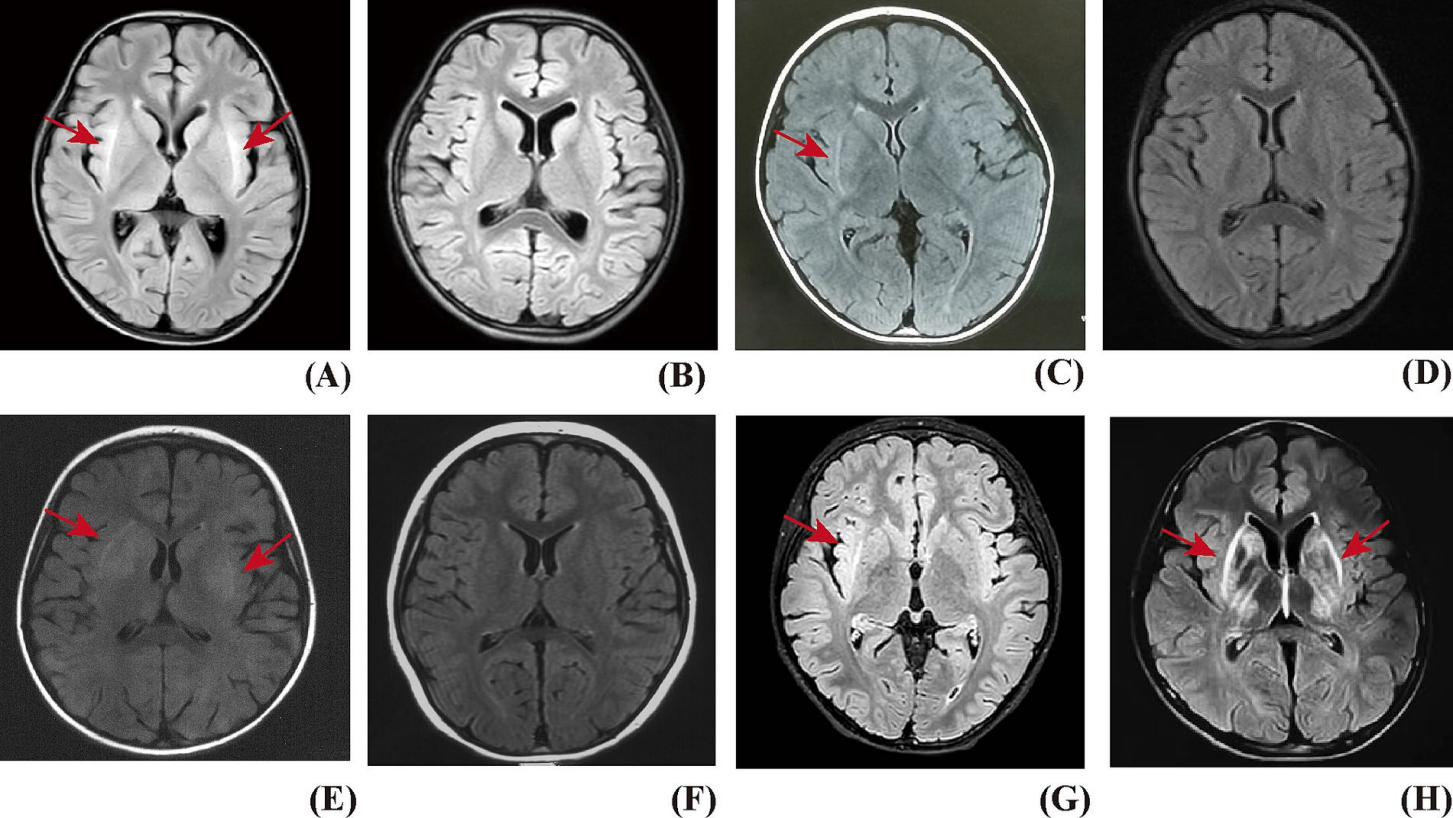
The claustrum sign on T2 FLAIRE of brain MRI. **(A)** Patient with FIRES, bilateral claustrum hyperintensity presented on MRI. **(B)** The claustrum sign disappeared on the follow-up MRI. **(C)** Patient with AbNAE, right claustrum hyperintensity presented on MRI, with co-occurring lesions in the head of the caudate nucleus and the putamen. **(D)** The claustrum sign disappeared on the follow-up MRI. **(E)** Patient with MOGAD, bilateral claustrum hyperintensity presented on MRI, with co-occurring lesions in the bilateral head of the caudate nucleus, putamen, and thalamus. **(F)** The claustrum sign disappeared on the follow-up MRI. **(G)** Patient with AbNAE, showed right claustrum hyperintensity presented on MRI, which persisted on the follow-up MRI on Day 640. **(H)** Patient with WD, bilateral claustrum hyperintensity presented on MRI, with co-occurring lesions in the head of the caudate nucleus and the putamen, which persisted on the follow-up MRI on Day 162.

**Figure 2:**
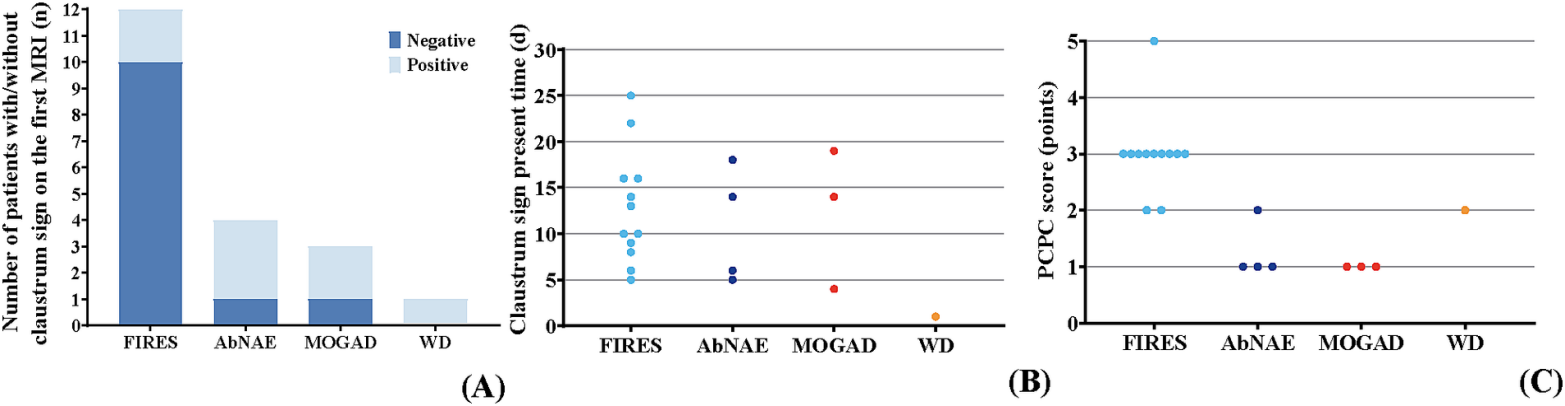
Evolution in the claustrum sign and prognosis in different diseases. **(A)** The number of patients with negative/positive claustrum sign in the first brain MRI among different diseases. In the FIRES group (*n* = 12), two patients showed the claustrum sign on the first MRI. It was negative on the first MRI in the remaining 10 patients. In the AbNAE group (*n* = 4), three patients showed the claustrum sign on the first MRI. The claustrum sign was negative on the first MRI in another patient. In the MOGAD group (*n* = 3), two patients showed the claustrum sign on the first MRI. The claustrum sign was negative on the first MRI in another patient. The claustrum sign was shown on the first MRI in the patient with WD. **(B)** The timing of the claustrum sign among different diseases. The claustrum sign was observed on days 5–25 after the onset of neurologic symptoms in patients with FIRES. The claustrum sign was observed on days 5–18 after the onset of neurologic symptoms in patients with AbNAE. The claustrum sign was observed on days 4–19 after the onset of neurologic symptoms in patients with MOGAD. The claustrum sign was observed on the first day after the onset of neurologic symptoms in the patient with WD. **(C)** The PCPC score among different diseases. In the FIRES group, the PCPC scores ranged from 2 to 5, with most patients having a PCPC score of 3. In the AbNAE group, the PCPC scores ranged from 1 to 2, with most patients having a PCPC score of 1. The PCPC scores were all 1 in the MOGAD group. The PCPC score was 2 in the patient with WD.

In 20 patients, the claustrum sign was observed on days 1–25 (median: day 11.5) after the onset of neurologic symptoms. It presented on the first MRI between days 1 and 14 (median: day 5.5) in 8 patients (40%), while it was negative on the first MRI (days 1–7, median: day 3) in the remaining 12 patients (60%) and appeared on the repeated MRIs (days 6–25, median: day 15) (Figure 1). Bilateral claustrum involvement occurred in 14 patients (70%), unilateral in 6 patients (30%). Two (10%) patients had isolated claustrum lesions, while in the other 18 patients (90%), claustrum lesions were accompanied by insula (45%), hippocampus (35%), frontal lobe (30%), head of caudate nucleus (30%), putamen (25%), temporal lobe (25%), thalamus (20%), parietal lobe (15%), occipital lobe (10%), and brainstem (10%) (Table 1 and Figure 2).

**Table 1:** Neuroimaging features of the claustrum sign.

No	Diagnosis	G	Age of onset (years)	First MRI time (d)	Claustrum sign present time (d)	Claustrum sign resolution time (d)	Concomitant lesions
1	FIRES	M	4.1	D3	D8	D88	Insula, hippocampus
2	FIRES	F	8.4	D7	D16	D34	Hippocampus
3	FIRES	M	11.9	D7	D25	D58	Hippocampus, insula, temporal lobe, frontal lobe, parietal lobe, thalamus
4	FIRES	M	4.9	D1	D6	D74	Hippocampus, head of caudate nucleus, putamen
5	FIRES	F	7.4	D5	D5	D53	Insula, frontal lobe, temporal lobe, parietal lobe, hippocampus, head of caudate nucleus
6	FIRES	F	11.2	D2	D13	D57	Insula
7	FIRES	M	8.6	D4	D16	D95	Temporal lobe, hippocampus
8	FIRES	M	4.0	D5	D22	D46	Hippocampus
9	FIRES	M	5.9	D3	D10	D52	N
10	FIRES	F	6.3	D2	D14	D55	Frontal lobe, temporal lobe, insula, occipital lobe
11	FIRES	M	5.8	D10	D10	NA	Frontal lobe, temporal lobe, insula
12	FIRES	M	6.8	D3	D9	D47	Insula
13	AbNAE	M	4.2	D2	D18	D71	Frontal lobe
14	AbNAE	M	11.2	D5	D5	D29	Insula
15	AbNAE	F	3.2	D6	D6	D37	Head of caudate nucleus, putamen
16	AbNAE	M	5.4	D14	D14	Persistent	N
17	MOGAD	F	4.1	D14	D14	D38	Head of caudate nucleus, putamen, parietal lobe, thalamus
18	MOGAD	M	5.5	D1	D19	D132	Frontal lobe
19	MOGAD	M	6.1	D4	D4	D16	Insula, thalamus, head of caudate nucleus, putamen
20	WD	M	12.6	D1	D1	Persistent	Brainstem, head of caudate nucleus, putamen, thalamus

FIRES, febrile infection-related epilepsy syndrome; AbNAE, antibody-negative autoimmune encephalitis; MOGAD, myelin oligodendrocyte glycoprotein antibody-associated disease; WD, Wilson’s disease; G, gender; M, male; F, female; D, day; NA, not available; N, none.

### 3.3 Disease spectrum and clinical manifestation

Among the 20 patients, 12 (60%) were diagnosed with FIRES, 4 (20%) with autoantibody-negative autoimmune encephalitis (AbNAE), 3 (15%) with myelin oligodendrocyte glycoprotein antibody-associated disease (MOGAD), and 1 (5%) with WD. Except for the WD patient, 19 patients (95%) had febrile infections preceding or concurrent with the onset of neurological symptoms. The neurological manifestations comprised seizures in 17 patients (85%), impaired consciousness in 14 patients (70%), psychiatric symptoms in 10 patients (50%), speech dysfunction in 5 patients (25%), movement disorders in 3 patients (15%), and headache in 2 patients (10%) (Table 2).

**Table 2:** Clinical characteristics of the claustrum sign.

No	Diagnosis	Fever	Clinical manifestation	CSF WBC cells	CSF Protein (mg/L)	CSF inflammatory factor (mg/L)	Autoimmune antibody testing	EEG
1	FIRES	Y	Seizures, impaired consciousness	13	570	NA	Negative	Slow waves, frequent seizures, migratory
2	FIRES	Y	Seizures, impaired consciousness, psychiatric symptoms	47	Normal	Normal	Negative	Slow waves, frequent seizures, migratory
3	FIRES	Y	Seizures, impaired consciousness	Normal	756	NA	Negative	Slow waves, frequent seizures
4	FIRES	Y	Seizures, impaired consciousness	78	1,500	IL-6 1705, IL-8 468	Negative	Slow waves, frequent seizures
5	FIRES	Y	Seizures, impaired consciousness	11	Normal	NA	Negative	Slow waves, frequent seizures
6	FIRES	Y	Seizures, impaired consciousness, psychiatric symptoms	Normal	Normal	NA	Negative	Slow waves, frequent seizures
7	FIRES	Y	Seizures, impaired consciousness, psychiatric symptoms	Normal	Normal	NA	Negative	Slow waves, frequent seizures, migratory
8	FIRES	Y	Seizures, impaired consciousness, psychiatric symptoms	30	Normal	NA	Negative	Slow waves, frequent seizures
9	FIRES	Y	Seizures, impaired consciousness, psychiatric symptoms	Normal	Normal	IL-6 133, IL-8 1810	Negative	Slow waves, frequent seizures
10	FIRES	Y	Seizures, impaired consciousness, psychiatric symptoms, speech dysfunction	28	Normal	IL-6 356, IL-8 865	Negative	Slow waves, frequent seizures, migratory
11	FIRES	Y	Seizures, impaired consciousness, psychiatric symptoms	14	Normal	NA	Negative	Slow waves, frequent seizures
12	FIRES	Y	Seizures, impaired consciousness, psychiatric symptoms	6	Normal	NA	Negative	Slow waves, frequent seizures, migratory
13	AbNAE	Y	Seizures, impaired consciousness	34	Normal	Normal	Negative	Slow waves
14	AbNAE	Y	Seizures, movement disorders	88	580	IL-6 202, IL-8 160	Negative	Slow waves
15	AbNAE	Y	Seizures, psychiatric symptoms, headache	64	2,300	NA	Negative	Slow waves
16	AbNAE	Y	Movement disorders, speech dysfunction	Normal	Normal	NA	Negative	Slow waves
17	MOGAD	Y	Seizures, headache	202	Normal	NA	MOG-IgG (1:1000)	Slow waves
18	MOGAD	Y	Seizures, psychiatric symptoms, speech dysfunction	8	Normal	NA	MOG-IgG (1:10)	Slow waves
19	MOGAD	Y	Impaired consciousness, speech dysfunction	8	Normal	NA	MOG-IgG (1:32)	Slow waves
20	WD	N	Movement disorders, speech dysfunction	Normal	Normal	NA	Negative	Normal

FIRES, febrile infection-related epilepsy syndrome; AbNAE, antibody-negative autoimmune encephalitis; MOGAD, myelin oligodendrocyte glycoprotein antibody-associated disease; WD, Wilson’s disease; Y, yes; N, no; NA not available.

### 3.4 Temporal relationship between seizures and claustrum sign

Among the 17 patients (85%) with seizures, the claustrum sign was observed on days 5–25 (median: day 13) after the onset of seizure. It presented on the first MRI between days 5 and 14 (median: day 6) in 5 patients (29%). It was negative on the first MRI (days 1–7, median: day 3) in the remaining 12 patients (71%) and appeared on the repeated MRIs (days 6–25, median: day 15). In the group of FIRES, all 12 patients experienced seizures from days 4 to 8 (median: day 6) after the onset of fever, and rapidly progressed to status epilepticus (SE). The claustrum sign was observed on days 5–25 (median: day 11.5) after the onset of seizure. Only 2 patients had the sign on the first MRI on day 5 and day 10, respectively. It was negative on the first MRI (days 1–7, median: day 3) in the remaining 10 patients, and which subsequently appeared on the repeated MRIs (days 6–25, median: day 13.5). In the group of AbNAE (*n* = 4), 3 patients (75%) experienced seizures on days 6–8 after the onset of fever. The claustrum sign appeared on the first MRI on day 5 and day 6 in 2 patients. In another one, the claustrum sign was absent on the first MRI (day 2) but appeared on the repeated MRI (day 18). In the group of MOGAD (*n* = 3), 2 patients (66.7%) experienced seizures on days 4–5 after the onset of fever. The claustrum sign presented on the first MRI on day 14. In the other one, it was negative on the first MRI (day 1), but was observed on the repeated MRI (day 19).

### 3.5 Temporal relationship between impaired consciousness and claustrum sign

Fourteen patients (70%) had a decline in consciousness. The claustrum sign was observed on days 4–25 (median: day 11.5) after the onset of impaired consciousness. It appeared on the first MRI between days 4 and 10 (median: day 5) in 3 patients (21%). In the remaining 11 patients (79%), it was negative on the first MRI (days 1–7, median: day 3) but was detected on the repeated MRIs (days 6–25, median: day 14). In the group of FIRES, all 12 patients developed impaired consciousness concurrently with seizures. The temporal relationship between impaired consciousness and claustrum sign was the same as that between seizures and claustrum sign. In the group of AbNAE group (*n* = 4), only 1 patient (25%) manifested impaired consciousness on day 8 after the onset of fever. The claustrum sign was absent on the first MRI (day 2) but observed on the repeated MRI (day 18). In the group of MOGAD (*n* = 3), only 1 patient (33.3%) experienced impaired consciousness on day 3 after the onset of fever. The claustrum sign was detected on day 4 after the onset of impaired consciousness.

### 3.6 Therapeutic response and imaging evolution

Excluding the WD patient, 19 (95%) patients received immunotherapies including: corticosteroids, high-dose intravenous immunoglobulin (IVIG), rituximab, mycophenolate mofetil, tocilizumab, anakinra, and plasma exchange. Antiseizure medications (ASMs) were used in 15 (75%) patients, ketogenic diet therapy in 3 (15%) patients, and vagus nerve stimulation (VNS) in 2 (10%) patients. One patient received deep brain stimulation (DBS) and anti-spasticity pharmacotherapy. The patient with WD was managed with low-copper diet and penicillamine.

The median follow-up time was 1.8 years (range: 0.9–4.3 years). Six patients (30%) achieved full recovery, while 14 patients (70%) developed neurological sequelae. During the follow-up phase, 19 patients underwent repeat MRI. The claustrum sign resolved in 17 patients between days 16 and 132 (median: day 53). It remained in one patient with WD and one patient with AbNAE, on day 162 and day 640, respectively. In the group of FIRES, all 12 patients (100%) developed chronic epilepsy, 9 patients (75%) showed poor memory and calculation, 1 patient retained impaired consciousness. The median PCPC score was 3 (range: 2–5). Of the 11 patients who had repeat MRIs, the claustrum sign resolved in all cases after remission of SE, despite persistent seizures in all cases and impaired consciousness in one case. In the group of AbNAE group 3 patients (75%) achieved full recovery, while 1 patient continued to experience movement disorders with maintained claustrum sign. The median PCPC score was 1 (range: 1–2). In the group of MOGAD, all 3 patients achieved full recovery with PCPC scores of 1 (Table 3).

**Table 3:** Treatment and prognosis in the claustrum sign-associated diseases.

No	Diagnosis	Immunotherapy	Other therapy	Follow time (year)	Outcome	PCPC score
1	FIRES	CS, IVIG	ASMs, VNS	2.5	Epilepsy, memory and calculation deficits	3
2	FIRES	CS, IVIG, RTX, TCZ	ASMs	1.8	Epilepsy, memory and calculation deficits	3
3	FIRES	CS, IVIG	ASMs, KD	2.3	Epilepsy, memory and calculation deficits	3
4	FIRES	CS, IVIG, TCZ, PLEX, anakinra	ASMs, KD	1.0	Epilepsy, impaired consciousness	5
5	FIRES	CS, IVIG	ASMs	0.9	Epilepsy, memory and calculation deficits	3
6	FIRES	CS, IVIG, TCZ	ASMs	1.3	Epilepsy, memory and calculation deficits	3
7	FIRES	CS, IVIG	ASMs	3.8	Epilepsy, memory and calculation deficits	3
8	FIRES	CS, IVIG, RTX, TCZ	ASMs, KD	1.5	Epilepsy	2
9	FIRES	CS, IVIG, TCZ	ASMs, VNS	1.3	Epilepsy	2
10	FIRES	CS, IVIG, MMF, TCZ	ASMs	2.9	Epilepsy, memory and calculation deficits	3
11	FIRES	CS, IVIG	ASMs	1.6	Epilepsy, memory and calculation deficits	3
12	FIRES	CS, IVIG, RTX, MMF, TCZ, PLEX, anakinra	ASMs	1.0	Epilepsy, memory and calculation deficits	3
13	AbNAE	CS, IVIG, RTX	ASMs	2.2	Normal	1
14	AbNAE	CS, IVIG	N	1.4	Normal	1
15	AbNAE	CS, IVIG, MMF	ASMs	4.3	Normal	1
16	AbNAE	CS, IVIG, MMF, TCZ	DBS, anti-spasticity pharmacotherapy	1.8	Movement disorders	2
17	MOGAD	CS, IVIG	N	2.2	Normal	1
18	MOGAD	CS, IVIG	ASMs	2.8	Normal	1
19	MOGAD	CS, IVIG, RTX	N	4.0	Normal	1
20	WD	N	Low-copper diet, penicillamine	1.3	Movement disorders	2

FIRES, febrile infection-related epilepsy syndrome; AbNAE, antibody-negative autoimmune encephalitis; MOGAD, myelin oligodendrocyte glycoprotein antibody-associated disease; WD, Wilson’s disease; CS, corticosteroids; IVIG, intravenous immunoglobulin; RTX, rituximab; MMF, mycophenolate mofetil; TCZ, tocilizumab; PLEX, plasma exchange; ASMs, antiseizure medications; VNS, vagus nerve stimulation; DBS, deep brain stimulation; KD, ketogenic diet; N, no.

## 4 Discussion

Claustrum, a thin gray matter structure between the insular cortex and the putamen, is enveloped and separated by the extreme capsule and external capsule of the deep cerebral white matter. There is one on each of the left and right sides, and it is one of the most mysterious structures in the brain (14). Composed predominantly of glutamatergic neurons with sparse GABAergic interneurons (15, 16), the claustrum demonstrates extensive glutamatergic axonal projections to the cerebral cortex (17, 18). Extensively interconnected with almost all cortical and subcortical regions, the claustrum is recognized as one of the most densely connected structures in the human brain relative to its small volume (19, 20). The claustrum is involved in numerous crucial brain functions, including consciousness formation, multisensory integration, sleep, attention, and memory storage (21). Alterations of this structure are implicated in Parkinson’s disease, Alzheimer’s disease, autism, schizophrenia, depression and WD. In recent years, the claustrum lesion—the claustrum sign has been frequently reported in neuroinflammatory diseases, including NORSE, AE/AAE, FIRES and ANE. Similar imaging lesions are often recognized as insular and external capsule lesions in the clinic. Nevertheless, its pathophysiological basis remains elusive. In this study, we have established a case cohort of the claustrum sign in children, which encompasses multiple disease types. The clinical and imaging features were analyzed in pediatric patients with claustrum sign, aiming to elucidate its evolutionary pattern and diagnostic implications.

In this case series, FIRES accounted for the majority of patients (60%), followed by AbNAE (20%), MOGAD (15%), and WD (5%), suggesting that the claustrum sign is more frequently associated with immune-mediated and inflammatory disorders. FIRES is a severe epileptic encephalopathy characterized by refractory status epilepticus with high mortality. Survivors often develop drug-resistant epilepsy and cognitive impairments. Emerging evidence classifies FIRES as an infection-triggered encephalopathy syndrome (ITES)-associated syndrome due to its similarities with ITES (22). Although the pathogenesis of ITES remains elusive, studies propose that peripheral infections may indirectly activate microglia and astrocytes in the central nervous system, triggering cytokine storms through excessive proinflammatory cytokine release. Existing evidence suggests that cytokine-mediated neuroinflammation plays a pivotal role in the initiation, maintenance, and progression of FIRES/ITES (23–25). The pathogenic mechanisms underlying AbNAE and MOGAD also involve autoimmune and cytotoxicity-mediated neuroinflammation (26). Excessive copper deposition in patients with WD can also lead to neuroinflammation, resulting in nerve damage (27). For these reasons, we hypothesize that the claustrum sign may serve as a neuroimaging biomarker of neuroinflammatory processes. Previous case reports documented that in a mumps virus encephalitis patient, the claustrum sign was observed on initial MRI on day 6 following the onset of seizure, potentially associated with post-infectious demyelination (28). In another patient with herpes simplex virus (HSV) encephalitis, the claustrum sign presented on the second MRI at day 10 after the onset of neurological symptoms. At that time, CSF HSV-DNA testing was negative (29). It is noteworthy that 95% of cases had antecedent febrile infections and no pathogens were detected in the CSF of all children in this cohort. These findings further suggest that the claustrum sign may be a result of neuroinflammatory responses triggered by viral or other pathogenic infections, rather than direct viral invasion-induced structural damage. The claustrum is rich in glutamatergic neurons, which may make this structure a susceptible target for neuroinflammation. Glutamate-mediated excitotoxicity could further exacerbate the cascade of neuroinflammatory damage. In this cohort the claustrum sign remained undetectable in 60% of cases undergoing MRI during the initial week of neurological symptoms, which demonstrates a temporal dissociation between the imaging feature and neurologic symptoms. This phenomenon might mirror the neuroinflammatory pathological process. Meanwhile, immunotherapies were clinically effective except for the WD patient. Among the 19 patients with MRI follow-up, the claustrum sign resolved during or after treatment in 17 patients (89%), except for the WD patient and one AbNAE patient presenting with movement disorders. Based on dynamic imaging evolution and therapeutic responses, these findings indicate that the claustrum sign may represent transient structural alterations in the claustrum secondary to neuroinflammatory processes, while persistent autoimmune-mediated damage or irreversible neuronal degeneration may contribute to sustained structural alterations.

Among the 20 patients in this study, the most prevalent clinical manifestation was seizures (85%, 17/20), followed by impaired consciousness (70%, 14/20), psychiatric symptoms (50%, 10/20), speech dysfunction (25%, 5/20) and a few presented with movement disorders (15%, 3/20) and with headache (10%, 2/20). These findings are consistent with the clinical manifestations of the claustrum sign reported in previous literature (30). The relationship between the claustrum sign and seizures remains unclear. Some studies suggest that the claustrum sign is an imaging consequence of neuronal damage caused by cytotoxic edema resulting from recurrent seizures (3, 31). Meletti et al. (4) documented the claustrum sign appearing 10 days post-SE in 31 cryptogenic NORSE patients (28 with prodromal fever). Among 12 patients with MRI follow-up, one showed persistent lesions, one resolved during SE, and the remaining 10 resolved within weeks after SE remission. Steriade et al. (6) reported the claustrum sign in two NORSE patients with prodromal fever, one anti-Ma2 antibody encephalitis patient with SE and one intractable autoimmune epilepsy patient. These positive imaging studies were acquired with a time lag of 10 days–4 months from the onset of seizure. The claustrum sign resolved in three patients in this study. Conversely, alternative hypotheses suggest the claustrum sign might contribute to seizures, even SE (32). In this cohort, 85% (17/20) of patients had seizures, the claustrum sign was observed on days 5–25 (median: day 13) after the onset of seizure. In the group of FIRES, all patients developed SE, the claustrum sign was absent on the first MRI in 10 patients (83%) within 1–7 days (median: day 3) after the onset of seizure. Notably, SE resolution preceded the disappearance of claustrum sign. However, seizures persisted in all cases after the resolution of claustrum sign. Three patients with AbNAE and two with MOGAD exhibited seizures without progression to SE. In two of them, the claustrum sign was not observed in the first MRI on days 1 to 2 after the onset of seizure. After the claustrum sign resolution, seizures persisted in only one AbNAE case. These findings demonstrate that the claustrum sign is neither a direct consequence of seizures nor a primary cause of seizures. In previous reports of post-coronavirus disease 2019 (COVID-19) encephalitis (33), anti-GluR encephalitis (34), and WD (2, 35, 36), there were also patients with the claustrum sign but no seizures, supporting our inference. In this cohort, 70% (14/20) of patients had impaired consciousness. The majority of those with impaired consciousness were accompanied by seizures, and only one patient with MOGAD did not have seizures. The claustrum sign was observed on days 4–25 (median: day 11.5) after the onset of impaired consciousness. In the group of FIRES, 11 cases recovered consciousness before the claustrum sign resolution. However, only 1 case continued to have impaired consciousness after the claustrum sign resolved. One patient with AbNAE and the other patient with MOGAD recovered consciousness before the disappearance of the claustrum sign. These findings suggest that there is no direct causal relationship between the claustrum sign and impaired consciousness. The claustrum’s neural circuitry is extensively connected to the cortex through glutamatergic neurons and modulates cortical synchronization by exciting or inhibiting the electrical activity of cortical interneurons (9, 37). Emerging evidence positions this structure as a pivotal hub in neural networks (38). The claustrum sign may not merely indicate structural abnormalities, but rather signify underlying network dysfunction that promotes seizure propagation and impairs consciousness modulation. Lesions in the claustrum can lead to dysfunction of its connected regions. This could be one of the potential mechanisms underlying the high incidence of seizures and consciousness impairment in children with the claustrum sign.

In this case series, children with FIRES all developed chronic epilepsy, 75% showed memory and calculation deficits, and the median PCPC score was 3. Among the children with AbNAE, 75% achieved full recovery with only one case having residual movement disorders, and the median PCPC score was 1. Children with MOGAD all achieved full recovery, and their PCPC scores were all 1. In terms of residual neurological sequelae and PCPC scores, children with FIRES had the poorest prognosis. This suggests that the long-term prognosis of children with the claustrum sign is closely related to the primary disease rather than the features of claustrum sign imaging.

Some scholars have proposed the claustrum sign as a diagnostic marker for FIRES (11). However, this imaging is not exclusively seen in FIRES. It has been reported in Anti-*N*-methyl-d-aspartate receptor (NMDAR) encephalitis (39), Anti-voltage-gated potassium channels (VGKC) encephalitis (40), Anti-glutamate receptor (GluR) encephalitis (34), Anti-contactin-associated protein-2 (CASPR2) encephalitis (41), Anti-glutamic acid decarboxylase 65 (GAD) encephalitis, Anti-Ma2 encephalitis and AbNAE (42). In our cohort, the claustrum sign occurred most frequently in FIRES, followed by AbNAE. Specifically, we observed this sign in 3 cases with MOGAD, which is the first report of the claustrum sign in MOGAD. These findings necessitate comprehensive diagnostic evaluations for patients presenting with seizures, consciousness impairment, and the claustrum signs, including autoimmune encephalitis antibody and CSF inflammatory markers (neopterin, quinolinic acid) to differentiate FIRES, AE, and MOGAD-associated cortical encephalitis. Clinicians should complete ceruloplasmin, 24-h urinary copper, and ATP7B genetic testing in patients with movement disorders and claustrum sign. The claustrum sign remained undetectable in 60% of cases undergoing MRI during the initial week of neurological symptoms in this cohort. For patients suspected of diseases such as FIRES, AE, and MOGAD, repeat brain MRI between 7 to 14 days after the onset of neurological symptoms is recommended, even if the claustrum sign is negative in the first MRI. This approach enhances the detection probability of the claustrum sign, a specific imaging biomarker for neuroinflammation. The identification of the claustrum sign may serve as critical evidence to prompt initiation or escalation of immunotherapy in the management of these conditions, thereby achieving improved clinical and imaging outcomes.

This study has several limitations. First, the single-center retrospective design resulted in a limited number of enrolled cases, the limited sample size constrains only descriptive analysis. Second, partial medical history information relied on clinical record review, which may introduce recall bias. Third, the time intervals between the appearance/disappearance of the claustrum sign and brain MRI may not fully capture the radiological evolution. Future prospective cohort studies with standardized longitudinal MRI follow-up are needed to better understand the dynamic evolution and pathophysiological significance of the claustrum sign. Additionally, it is also necessary to compare patients with and without the claustrum sign within the same disease to explore whether it can be used as an indicator of disease severity or for prognostic evaluation.

## 5 Conclusion

The claustrum sign is observed in FIRES, AbNAE, MOGAD, and WD. The predominant clinical manifestations are seizures and impaired consciousness. The claustrum sign may present unilaterally or bilaterally, frequently co-occurring with lesions in the insula, hippocampus, and other regions, most resolve during the clinical course. Long-term prognosis of children with this imaging feature correlates strongly with the primary disease. This imaging feature may represent a transient neuroinflammatory lesion and serve as an imaging marker of neuroinflammation. Enhancing understanding the clinical and neuroimaging characteristics of the claustrum sign can be helpful in the diagnosis and treatment of such disorders.

## Data Availability

The original contributions presented in the study are included in the article/supplementary material, further inquiries can be directed to the corresponding author.
